# Neuropeptide Y protects kidney from acute kidney injury by inactivating M1 macrophages via the Y1R-NF-κB-Mincle-dependent mechanism

**DOI:** 10.7150/ijbs.80200

**Published:** 2023-01-01

**Authors:** Rui-zhi Tan, Jian-chun Li, Bing-wen Zhu, Xiao-ru Huang, Hong-lian Wang, Jian Jia, Xia Zhong, Jian Liu, Li Wang, Hui-yao Lan

**Affiliations:** 1 Research Center of Intergated Traditional Chinese and Western Medicine, Affiliated Traditional Chinese Medicine Hospital, Southwest Medical University, Luzhou, 646000, China.; 2 Institute of Integrated Chinese and Western Medicine, Southwest Medical University, Luzhou, 646000, China.; 3 Department of Medicine and Therapeutics, Li Ka Shing Institute of Health Sciences, Lui Che Woo Institute of Innovative Medicine, the Chinese University of Hong Kong, Hong Kong, China.; 4 Guangdong-Hong Kong Joint Laboratory for Immunological and Genetic Kidney Disease, Guangdong Academy of Medical Science, Guangdong Provincial People's Hospital, Guangzhou, 510080, China.

**Keywords:** Neuropeptide Y, Y1R, AKI, Inflammation, Macrophage, Mincle

## Abstract

Neuropeptide Y (NPY) is produced by the nerve system and may contribute to the progression of CKD. The present study found the new protective role for NPY in AKI in both patients and animal models. Interestingly, NPY was constitutively expressed in blood and resident kidney macrophages by co-expressing NPY and CD68+ markers, which was lost in patients and mice with AKI-induced by cisplatin. Unexpectedly, NPY was renoprotective in AKI as mice lacking NPY developed worse renal necroinflammation and renal dysfunction in cisplatin and ischemic-induced AKI. Importantly, NPY was also a therapeutic agent for AKI because treatment with exogenous NPY dose-dependently inhibited cisplatin-induced AKI. Mechanistically, NPY protected kidney from AKI by inactivating M1 macrophages via the Y1R-NF-κB-Mincle-dependent mechanism as deleting or silencing NPY decreased Y1R but increased NF-κB-Mincle-mediated M1macrophage activation and renal necroinflammation, which were reversed by addition of NPY or by silencing Mincle but promoted by blocking Y1R with BIBP 3226. Thus, NPY is renoprotective and may be a novel therapeutic agent for AKI. NPY may act via Y1R to protect kidney from AKI by blocking NF-κB-Mincle-mediated M1 macrophage activation and renal necroinflammation.

## Introduction

Acute kidney injury (AKI) is defined as the sudden loss of kidney function due to the severe damage to the kidney, particularly to tubular epithelial cells. Increasing evidence shows that AKI is a major cause of chronic kidney disease (CKD) with high mortality 1-3. However, effective treatments for AKI remain limited and studies on mechanisms of AKI remain challenging.

There are many risk factors for AKI, including hypertension, ischemic injury, nephrotoxic drugs, sepsis, major surgery, etc. 1-3. Growing evidence shows that immune cells such as macrophages, dendritic cells, T cells, and neutrophils also participate in the pathogenesis of AKI 4-6. Among them, macrophages with the M1 phenotype play a critical role in the initiation and progression of AKI 7-14. In contrast, M2 macrophages may be renoprotective in AKI and determine the long-term outcomes of AKI by exhibiting their reparative or pro-fibrotic functions 14-18. Studies have shown that systematic removal of macrophages in mice can significantly alleviate AKI 19, 20. However, macrophage deletion may also remove the M2 macrophage with repairing function, which is detrimental to the repair process after AKI. Thus, directly deleting macrophages may not be a good therapeutic approach for AKI. The identification of specific mechanisms and development of more specific targeting therapies for inhibiting the proinflammatory activities of macrophages may be a better therapeutic strategy for AKI.

Neuropeptide Y (NPY), a 36-aa peptide, is a member of the NPY family including NPY, peptide YY (PYY), and pancreatic polypeptide (PP) 21. NPY is produced by the central and peripheral nervous system, as well as by immune cells including macrophages, and dendritic cells 22. Up to now, seven NPY receptor (YR) subtypes (Y1R, Y2R, Y4R, Y5R, Y6R-Y8R) have been described in vertebrates 23, 24. Among them, Y1R, Y2R, Y4R, and Y5R have functions in humans, whereas, Y3R has now been characterized as CXC chemokine receptor type 4 and is therefore included in the chemokine receptor family 24. NPY and its receptors have been reported to be involved in a variety of physiological activities, including regulation of brain activity, blood pressure, vessel constriction, food intake, energy balance, anxiety, body metabolism and immune functions 21-24. It is widely accepted that NPY is a risk factor in chronic cardiovascular disease 24, 25. Similarly, patients with chronic kidney disease (CKD) also have elevated circulating NPY, which correlates with severe renal dysfunction including proteinuria and declined glomerular filtration rate 24, 26, 27. It is also reported that NPY gene polymorphism in CKD patients is associated with the development of hypertension 28. Furthermore, increased serum NPY levels can predict a rapid progression of kidney injury and the cardiovascular events in patients with CKD 24, 29, 30. In contrast, it has been reported that NPY and its receptor Y1 are expressed by tubular epithelial cells in the normal kidney but decreased in the hypertensive kidney disease in rat 31. A recent study also found that NPY is expressed by mononuclear blood leukocytes, presumably by monocytes/macrophages, which is downregulated during acute renal allograft rejection 32. Interestingly, administration of NPY can protect kidney from cisplatin-induced acute kidney injury (AKI) by blocking p53-mediated cell apoptosis or improving bone marrow dysfunction 33, 34. However, the functional role of NPY in AKI remains unexplored, which was examined in the present study in cisplatin-induced mouse model of AKI in NPY knockout (KO) mice. The therapeutic effect and signaling mechanism of NPY in AKI were also examined *in vivo* and *in vitro*.

## Results

### NPY is produced by normal macrophages, which is lost when macrophages become activated with M1 phenotype in patients and mice with AKI

**Figure 1:**
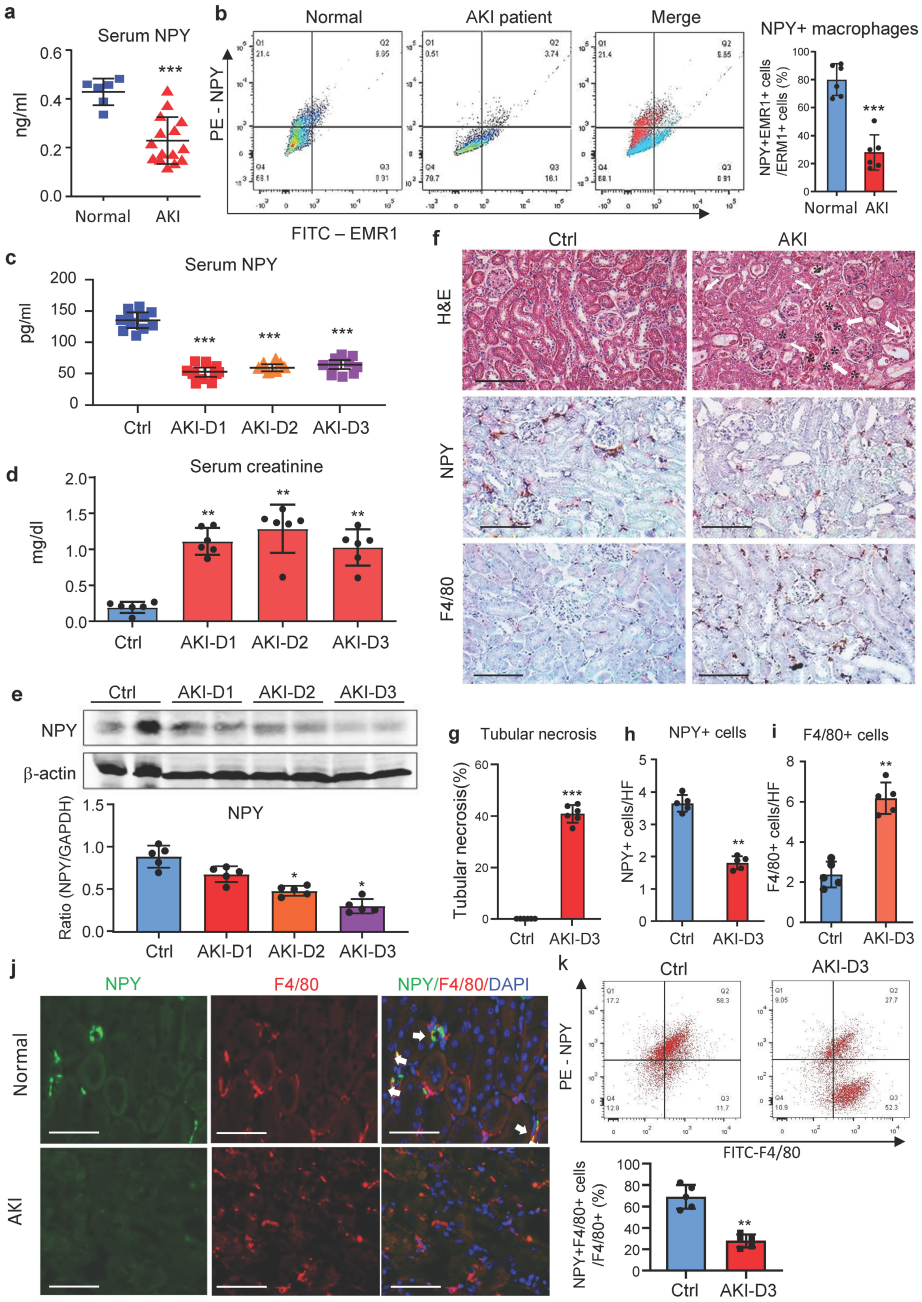
** NPY is produced by macrophages but rapidly decreased in patients and mice with AKI. a.** Serum levels of NPY by ELISA. Each dot represents one health individual or AKI patient. **b.** NPY-expressing macrophages (NPY+EMER1+) in the blood by flow cytometry. Each dot represents one health or AKI patient, and each bar is the mean ± SEM for groups of health people (n=6) or AKI patients (n=15). **c.** Serum levels of NPY from normal or AKI mouse. **d.** Serum levels of creatinine. **e**. Expression of renal NPY by western blotting. **f.** Tubular necrosis (*) by H&E staining, and NPY expression and F4/80+ macrophage infiltration in day 3 AKI kidneys by immunohistochemistry**. g-i.** Quantitative analysis of tubular necrosis, NPY expression, and F4/80+ macrophage infiltration in day 3 AKI kidneys. **J.** Tow-color immunofluorescence reveals NPY (green)-expressing F4/80+ macrophages (red) in the normal kidney are lost in the AKI kidney on day 3. **k.** Two-color flow cytometry detects a marked decrease in NPY-expressing macrophages in the AKI kidney on day 3. Each dot represents one mouse, and each bar is the mean ± SEM for groups of 5-8 mice (c-k). ^*^P<0.05*,* ^**^P<0.01*,* ^***^P<0.001* vs.* Ctrl group. Scale bar, 100 μm.

We first examined blood NPY levels in health people and patients with AKI by ELISA and found that compared to heath individuals, serum levels of NPY were significantly decreased in patients with AKI (Figure 1a), which was associated with a significant increase in serum creatine and the decline in glomerular filtration rate (Supplementary Table 1). Interestingly, two-color flow cytometry and immunofluorescence detected that NPY was highly expressed by blood and urinary monocytes/macrophages in heath people but was largely reduced in patients with AKI (Figure 1b and Supplementary Figure 1). Like AKI patients, serum levels of NPY in cisplatin-induced AKI mice were also rapidly decreased (Figure 1c), which was inversely associated with an increase in serum creatinine and the development of severe tubular necrosis (Figure 1d, f, g). Western blot and real-time PCR also detected that renal NPY expression in both mRNA and protein levels was largely reduced in the AKI kidney in a time-dependent manner, which was inversely associated in upregulation of M1 macrophage markers such as iNOS and Mincle and proinflammatory cytokines including IL-1β, TNFα, IL-6, MCP-1 (Figure 1e and Supplementary Figure 2). By immunohistochemistry, renal NPY was highly expressed by mononuclear cells infiltrating the tubulointerstitium in the normal mouse kidney, which was also largely lost in AKI kidneys with profound F4/80+ macrophage infiltration (Figure 1f, h, i). Two-color immunofluorescence and flow cytometry confirmed these notions and revealed that about 70-80% of NPY-expressing cells in the normal mouse kidney were macrophage origin by co-expressing NPY and F4/80 markers, which was also largely decreased in the AKI kidney (Figure 1j, k). These results indicate that most F4/80 positive cells express NPY in normal kidney.

### NPY plays a protective role in cisplatin-induced AKI in mice

**Figure 2:**
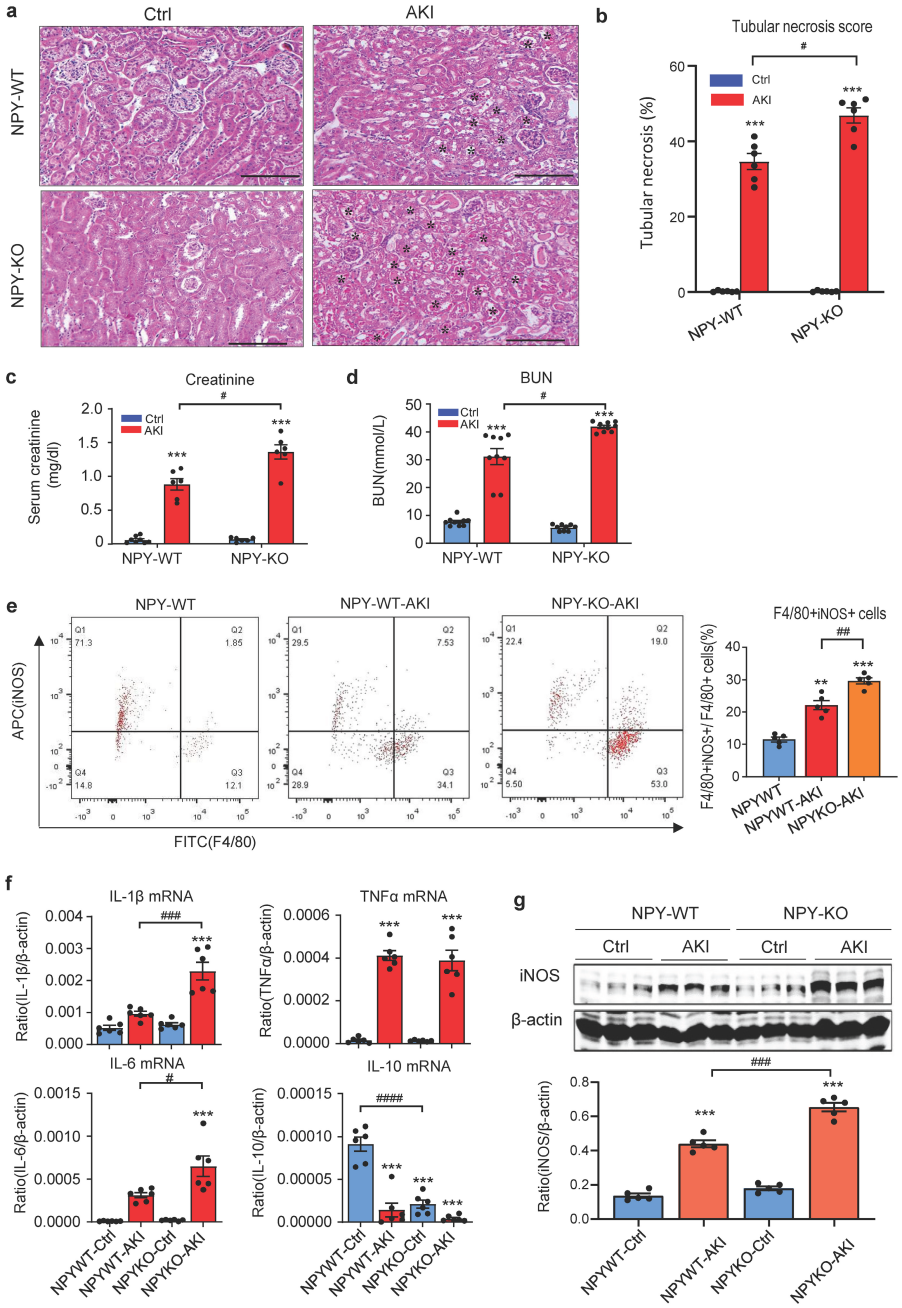
** Mice lacking NPY develop much more severe AKI induced by cisplatin. a.** H&E staining shows that mice lacking NPY are largely promoted tubular necrosis (*). **b.** Quantitative analysis of tubular necrosis. **c, d.** Serum creatinine and blood urea nitrogen (BUN). **e.** Renal iNOS+F4/80+ M1 macrophages detected by two-color flow cytometry. **f.** Renal mRNA expression of proinflammatory cytokines (IL-1β, IL-6, TNF-α and IL-10) by real-time PCR. **g.** Western blot analysis of renal iNOS expression. Each dot represents one mouse, and each bar is the mean ± SEM for groups of 6 mice. ^*^P<0.05*,* ^**^P<0.01*,* ^***^P<0.001* vs.* Ctrl group; ^#^*P*<0.05*,* ^##^*P*<0.01*,* ^###^*P*<0.001 as indicated. Scale bar, 100 μm.

To explore the role of NPY in AKI, we induced AKI in NPY WT and KO mice by injecting cisplatin (20mg/kg) i.p as previously described 9-11. Unexpectedly, compared to NPY WT mice in which severe AKI was developed with massive tubular necrosis and high levels of serum creatinine and BUN, mice lacking NPY developed worse AKI with a further increase in tubular necrosis and serum creatinine and BUN (Figure 2 a-d). Tow-color flow cytometry revealed that mice lacking NPY exhibited more severe renal inflammation as demonstrated by a significant increase in F4/80+iNOS+ M1 macrophages (Figure 2e) and a marked upregulation of proinflammatory cytokines such as IL-1β, IL-6, and iNOS as determined by western blots and real-time PCR (Figure 2f, g).

**Figure 3:**
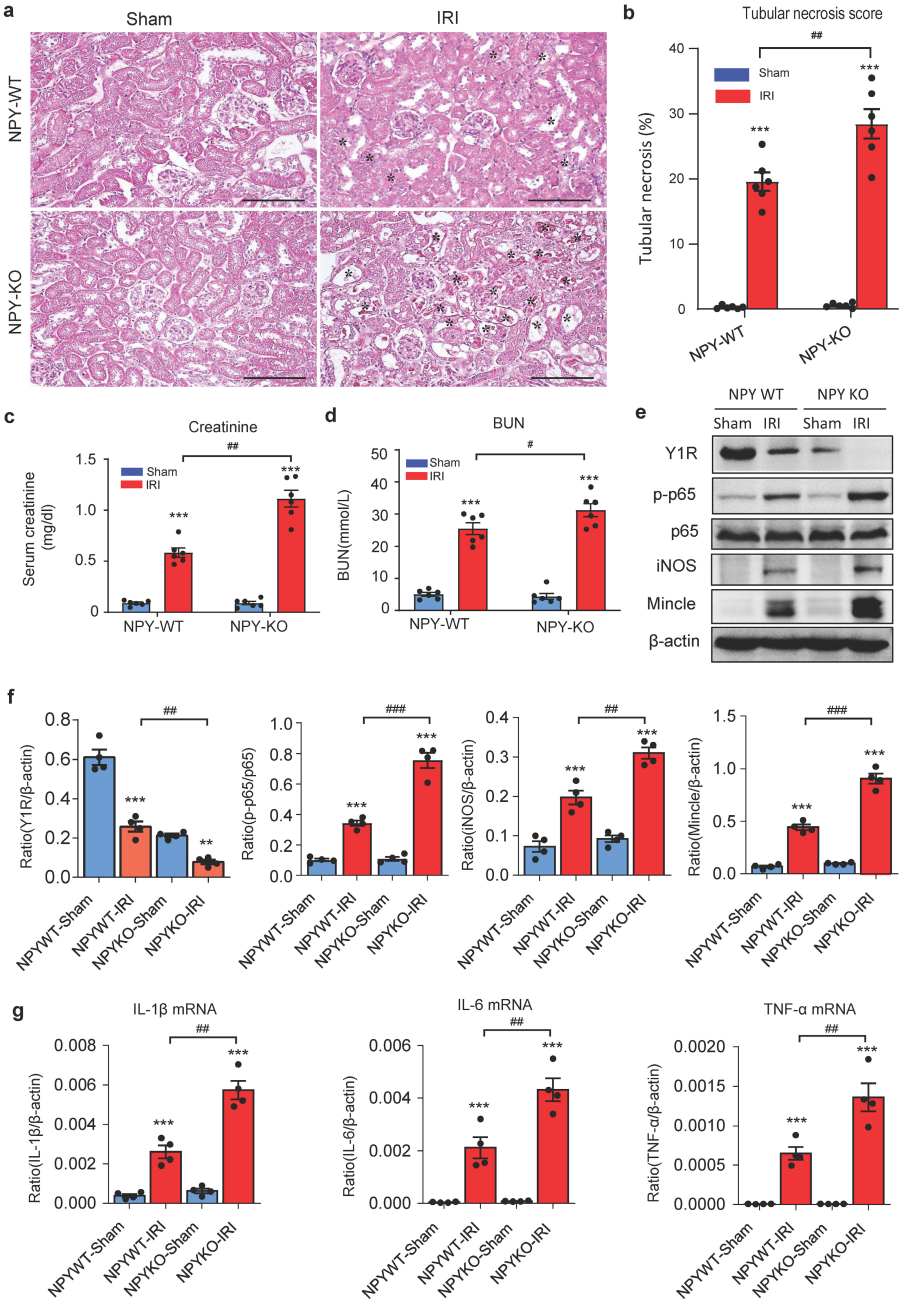
** Mice lacking NPY develop much more severe AKI induced by IRI. a.** H&E staining shows that mice lacking NPY are largely promoted IRI-induced tubular necrosis (*). **b.** Quantitative analysis of tubular necrosis. **c, d.** Serum creatinine and blood urea nitrogen (BUN). **e f.** Western blot analysis of renal Y1R and p-p65, iNOS, Mincle expression. **g.** Renal mRNA expression of proinflammatory cytokines (IL-1β, IL-6 and TNF-α) by real-time PCR. ^*^P<0.05*,* ^**^P<0.01*,* ^***^P<0.001* vs.* Ctrl group; ^#^*P*<0.05*,* ^##^*P*<0.01*,* ^###^*P*<0.001 as indicated. Scale bar, 100 μm.

To detect the role of NPY in different AKI models, we established ischemia reperfusion injury (IRI) AKI model in NPY WT and KO mice by clamping renal arteries for 35 minutes. Similar to the findings obtained from cisplatin-induced AKI, mice lacking NPY also developed worse AKI demonstrated by a marked increase in tubular necrosis and serum levels of creatinine and BUN when compared to the WT mice (Figure 3a-d). Western blot and real-time PCR analysis also detected that deletion of NPY largely increased expression of inflammatory cytokines including iNOS, Mincle, IL-1β, IL-6 and TNF-α in IRI-induced AKI kidney (Figure 3 e-g). All these findings suggested that NPY can protect kidney from cisplatin-and ischemic-induced AKI by inhibiting renal inflammation, particularly M1 macrophage activation.

### Treatment with NPY protects against cisplatin-induced AKI in mice

**Figure 4:**
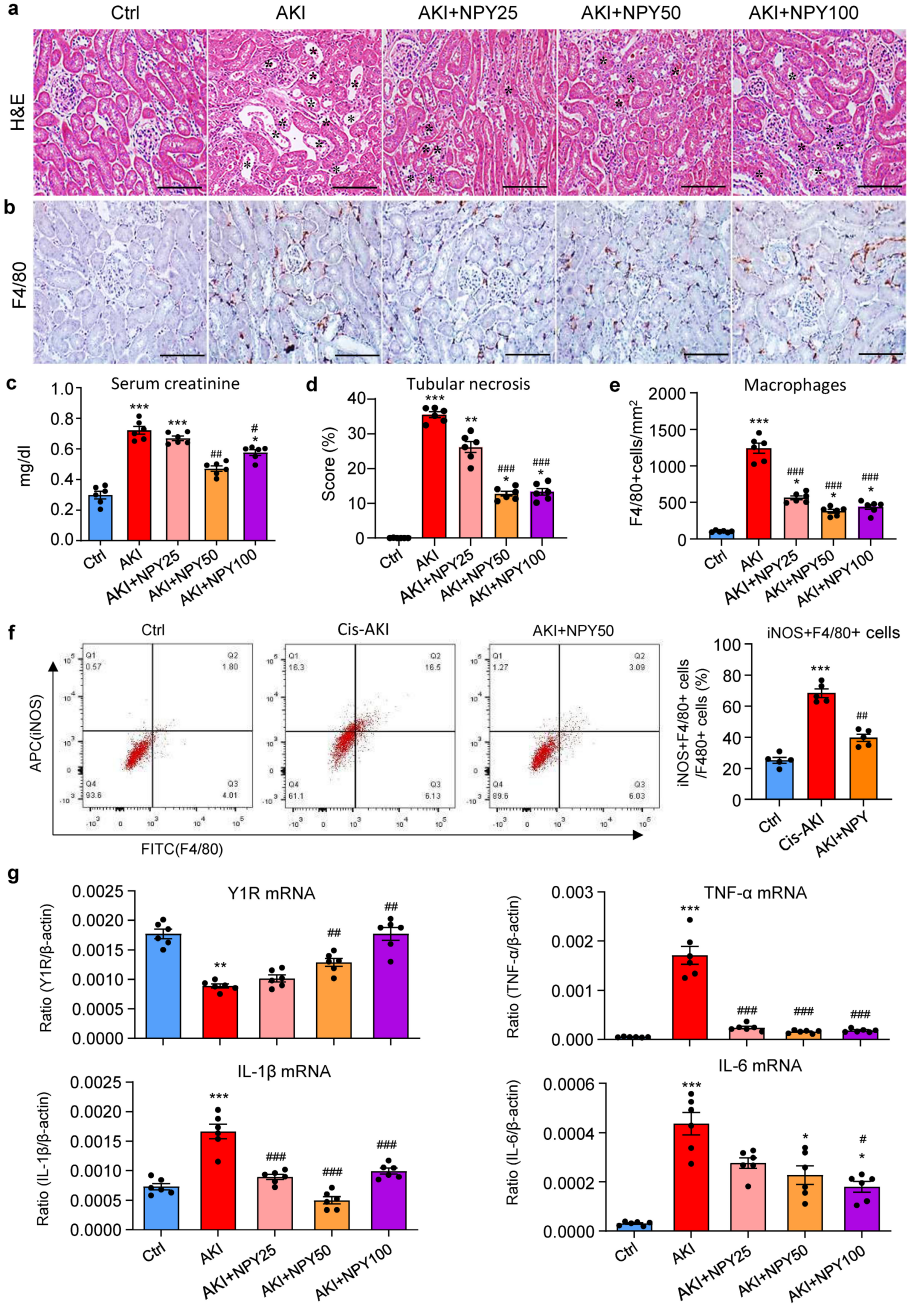
** Treatment with NPY protects against cisplatin-induced AKI in mice. a, d.** H&E staining shows that treatment with exogenous NPY dose-dependently inhibits cisplatin-induced tubular necrosis (*). **b, e.** Immunohistochemistry detects that treatment with exogenous NPY dose-dependently inhibits F4/80+ macrophage infiltration in the AKI kidney. **c.** serum levels of creatinine. **f.** Two-color flow cytometry detects that treatment with NPY (50 μg/kg/day) inhibits iNOS+F4/80+ M1 macrophages infiltrating the AKI kidney. **g.** Real-time PCR shows that treatment with exogenous NPY dose-dependently upregulated renal Y1R but inhibits expression of proinflammatory cytokines (IL-1β, IL-6 and TNF-α). Each dot represents one mouse, and data represent the mean ± SEM for groups of 6 mice. NPY25, NPY50 and NPY100 indicate that the dose of NPY treatment is 25, 50 and 100 μg/kg/day, respectively. ^*^P<0.05, ^**^P<0.01, ^***^P<0.001* vs.* Ctrl group; ^#^*P*<0.05, ^##^*P*<0.01, ^###^*P*<0.001* vs.* AKI without NPY treatment. Scale bar, 100 μm.

We next explored the therapeutic potential of NPY on AKI by treating NPY WT mice with exogenous NPY daily at dosages of 0, 25, 50, and 100 μg/kg body weight or saline control immediately after AKI induction for 3 days. We found that addition of NPY dose-dependently inhibited cisplatin-induced AKI by reducing the severity of tubular necrosis and serum levels of creatinine, with an optimal dose at 50 μg/kg/day (Figure 4 a, c, d). Interestingly, treatment with either a lower (25 μg/kg/day) or a higher (100 μg/kg/day) dose NPY did not produce more beneficial effect on AKI (Figure 4 a, c, d). Results from this study demonstrated that NPY has therapeutic effect on AKI with an optimal dose at 50 μg/kg/day.

As mice lacking NPY developed more severe AKI by promoting M1 macrophage activation and renal necroinflammation (Figures 2 and 3), we then examined whether the therapeutic effect of NPY on AKI is associated with inhibition of macrophage-mediated renal inflammation. As shown in Figure 4 (b, e), treatment with NPY dose-dependently inhibited F4/80+ macrophage infiltration, again with an optimal dose at 50 μg/kg/day. Two-color flow cytometry and real-time PCR also revealed that treatment with NPY dose-dependently inhibited M1 macrophage activation by reducing 50% F4/80+iNOS+ cells and largely suppressed expression of proinflammatory cytokines including IL-1β, IL-6 and TNFα in the AKI kidney (Figure 4f, g).

### NPY signals through Y1R to exert its protective effect on AKI by inactivating M1 macrophages via the NF-κB-Mincle-dependent mechanism *in vivo* and *in vitro*

**Figure 5:**
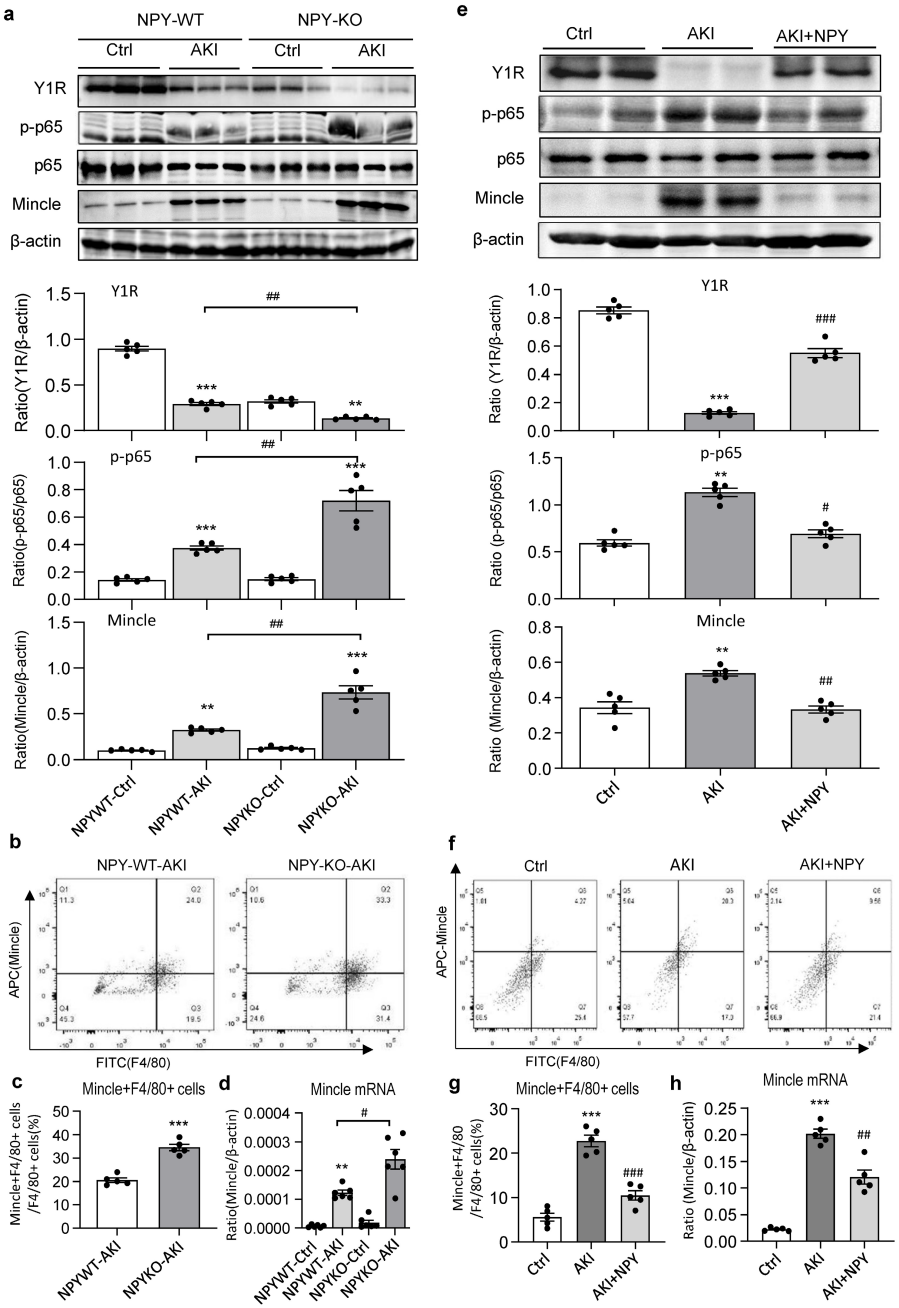
** Cisplatin-induced loss of Y1R while enhancing NF-κB-Mincle signaling and M1 macrophage activation in the AKI kidney is largely enhanced in NPY KO mice but inhibited by treatment with exogenous NPY. a**. Western blots reveal that cisplatin-induced AKI in NPY WT mice is associated with inhibition of Y1R but upregulation of NF-κB/p65-Mincle signaling, which is further enhanced in NPY KO mice. **b, c.** Two-color flow cytometry for Mincle+F4/80+ M1 macrophages infiltrating the AKI kidney of NPY WT or KO mice. **d.** Real-time PCR for renal Mincle mRNA expression in the AKI kidney of NPY WT or KO mice. **e**. Western blots show that treatment with NPY (50 μg/kg/day) for 3 days largely upregulates Y1R but blocks NF-κB/p65-Mincle signaling. **f, g.** Therapeutic effect of NPY on renal Mincle+F4/80+ M1 macrophages by two-color flow cytometry. **h.** Therapeutic effect of NPY on renal Mincle mRNA expression by real-time PCR. Each dot represents one mouse, and each bar is the mean ± SEM for groups of 5-6 mice. ^**^P<0.01, ^***^P<0.001* vs.* Ctrl group; ^#^*P*<0.05, ^##^*P*<0.01, ^###^*P*<0.001* vs.* AKI without treatment.

It is well established that the NF-κB-Mincle-Syk circuits is essential in triggering and maintaining M1 macrophage activation under AKI conditions 9-11. We next explored whether NPY can function as an immunoregulator to inactivate M1 macrophages via the Y1R-NF-κB-Mincle signaling mechanism in NPY WT/KO AKI mice. Analysis by western blots, two-color flow cytometry, and real-time PCR detected that cisplatin and ischemic-induced AKI in NPY WT mice was associated with downregulation of Y1R while upregulating NF-κB/p65-Mincle signaling and Mincle^+^F4/80^+^ M1 macrophages, which became much more severe in mice lacking NPY (Figure 3e and Figure 5a, b-d). In contrast, treatment of AKI mice with NPY largely increased Y1R but inhibited NF-κB-Mincle signaling, thereby blocking M1 macrophage activation (Mincle^+^iNOS^+^) in a dose-dependent manner (Figure 5 e-h and Supplementary Figure 3).

**Figure 6:**
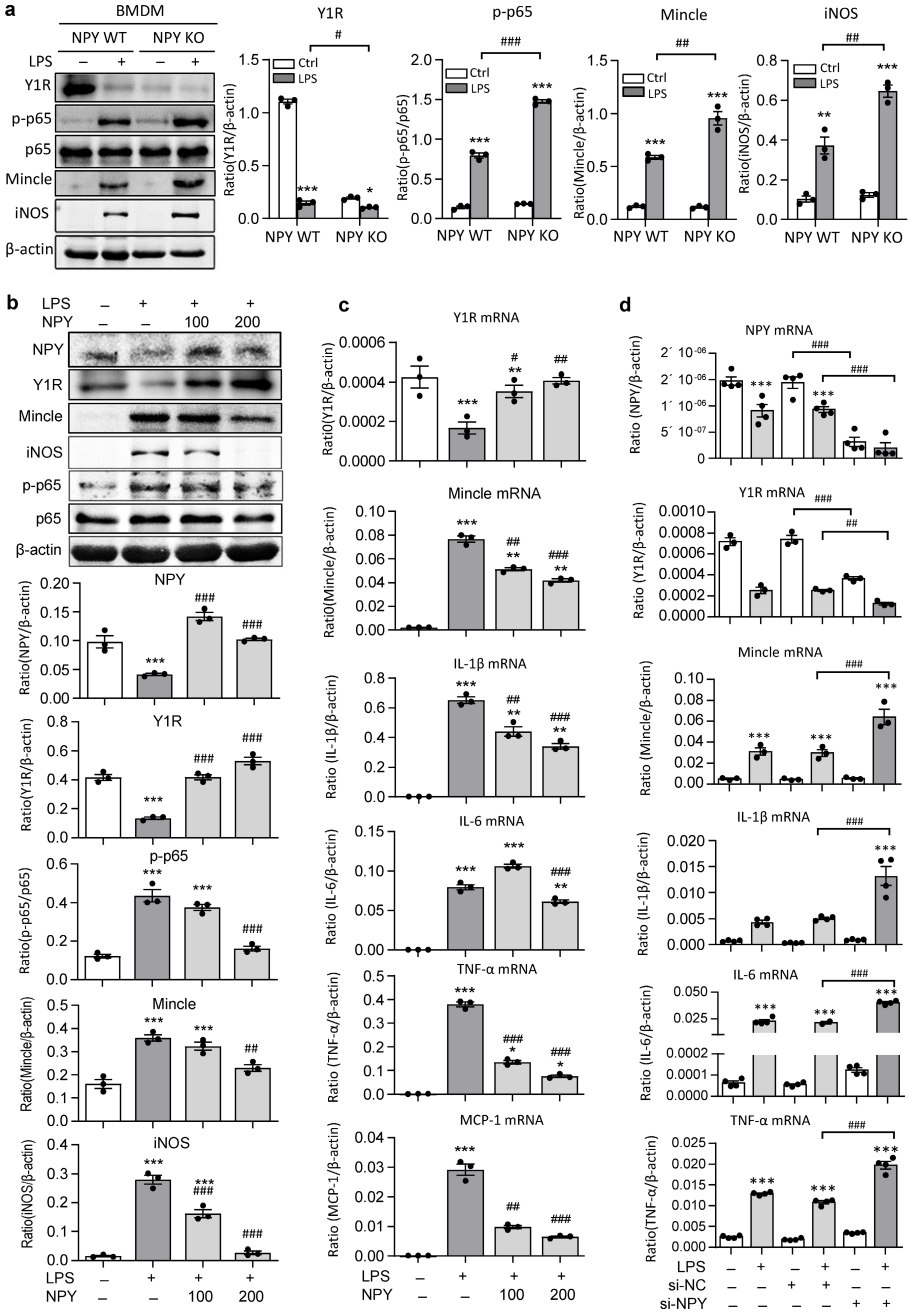
** BMDMs lacking NPY results in a loss of Y1R but largely enhances LPS-induced activation of NF-κB/p65-Mincle signaling and M1 macrophage activation *in vitro*. a b.** Western blots detect that BMDMs lacking NPY result in a loss of Y1R while largely promoting LPS (100ng/ml) -induced NF-κB/p65-Mincle signaling and activation of M1 macrophages (Mincle+ iNOS+), which are reversed by addition of NPY (0, 100, 200 mg/ml) in a dose-dependent manner. **c, d.** Real-time PCR shows that addition of NPY (0, 100, 200 mg/ml) to BMDMs dose-dependently inhibits LPS-induced loss of Y1R and upregulation of Mincle, IL-1β, IL-6, TNFα, and MCP-1 mRNA, which are largely reversed by silencing NPY. Each dot represents one independent experiment, and each bar is the mean ± SEM for 3-4 independent experiments. ^*^P<0.05, ^**^P<0.01, ^***^P<0.001* vs.* Ctrl group; ^#^*P*<0.05, ^##^*P*<0.01, ^###^*P*<0.001* vs.* LPS-stimulated group.

The regulatory role for NPY in inactivating M1 macrophages via the Y1R-NF-κB-Mincle-dependent mechanism was further investigated *in vitro* in BMDMs. We found that abundant cytoplasm NPY expression was detected in BMDMs, which was lost in response to LPS stimulation in a time-dependent manner (Supplementary Figure 4). Strikingly, compared to NPY WT cells, BMDMs lacking NPY were largely promoted LPS-induced inhibition of Y1R while enhancing NF-κB-Mincle signaling and a marked activation of M1 macrophages by co-expressing Mincle and iNOS (Figure 6a). In contrast, addition of NPY to BMDMs was able to block LPS-induced loss of NPY-Y1R signaling and activation of NF-κB/p65-Mincle signaling, thereby inhibiting M1 macrophage activation by blocking Mincle, iNOS, IL-1β, TNFα, IL-6, and MCP-1 expression in a dose-dependent manner (Figure 6b, c). Further studies in BMDMs also showed that silencing NPY inhibited NPY-Y1R signaling, thereby enhancing LPS-induced expression of Mincle, iNOS, IL-1β, TNFα and IL-6 by M1 macrophages (Figures 6d and Supplementary Figure 5). In contrast, silencing Mincle inhibited LPS-induced M1 macrophage activation (Supplementary Figure 5).

**Figure 7:**
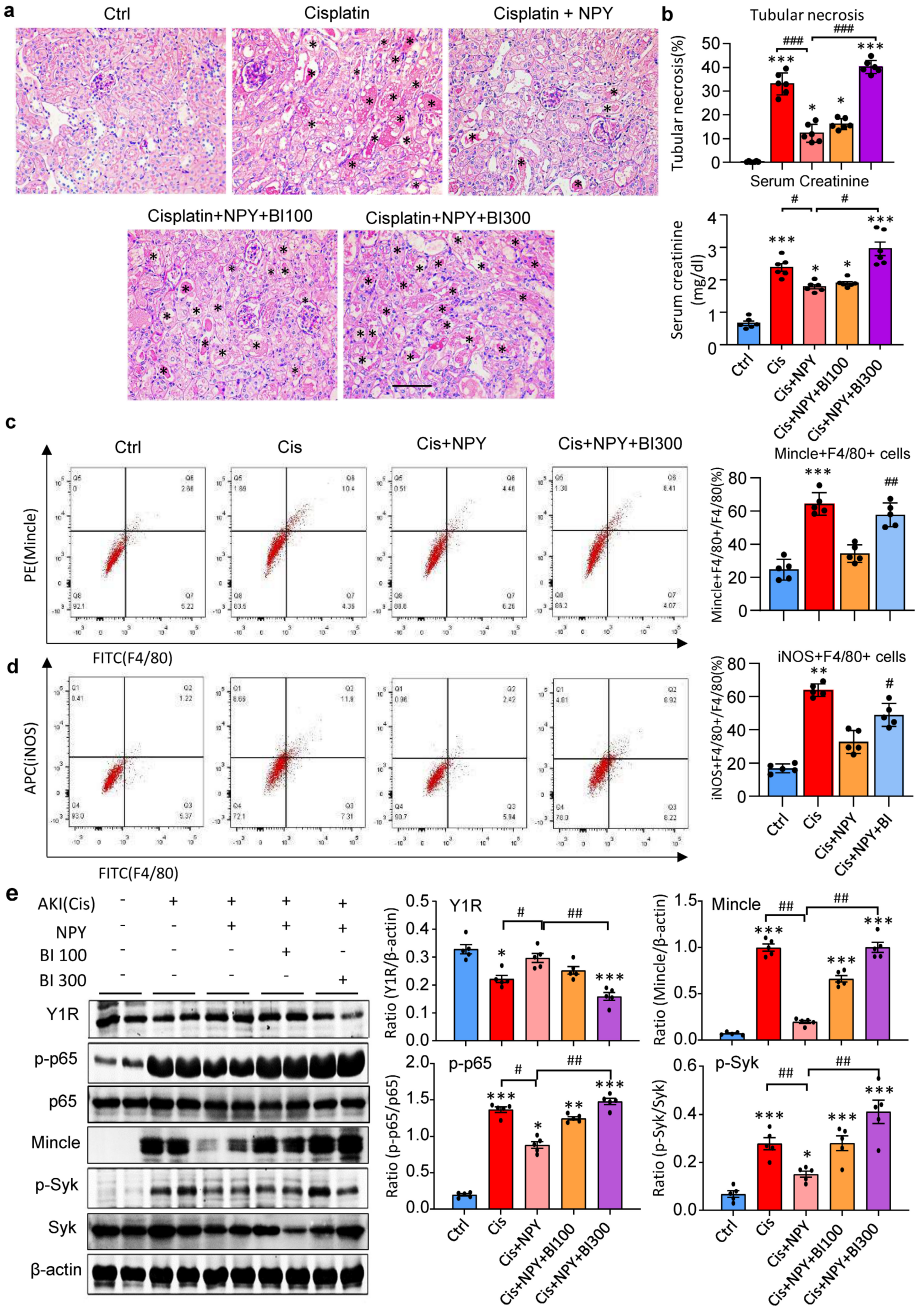
** Treatment with a Y1R antagonist BIBP 3226 abolishes the protective effect of NPY on cisplatin-induced AKI by inhibiting NPY-Y1R but largely enhancing NF-κB/p65-Mincle-Syk signaling in mice. a.** H&E staining detects that treatment with a Y1R antagonist BIBP3226 (0, 100, 300 mg/kg/day) dose-dependently blocks the renoprotective effect of NPY (50 μg/kg/day) on cisplatin-induced tubular necrosis (*). **b.** Quantitative analysis of tubular necrosis and** s**erum levels of creatinine**. c, d.** Two-color flow cytometry detects that treatment with a Y1R antagonist BIBP3226 (100 mg/kg/day) reverses the suppressive effect of NPY (50 μg/kg/day) on M1 macrophage (Mincle+iNOS+F4/80+) activation in cisplatin-induced AKI kidneys. **e**. Wester blots detect that treatment with BIBP 3226 (0, 100, 300 mg/kg/day) blocks NPY-Y1R but inhibits NF-κB/p65-Mincle-Syk signaling in the AKI kidney in a dose-dependent manner. Each dot represents one mouse, and data represent the mean ± SEM for groups of 5-6 mice. BI100 and BI300 indicate that the administration dose of BIBP3226 is 100 and 300 mg/kg/day, respectively. ^*^P<0.05, ^***^P<0.001* vs.* Ctrl group; ^#^*P*<0.05, ^##^*P*<0.01, ^###^*P*<0.001 as indicated. Scale bar, 50 μm.

**Figure 8:**
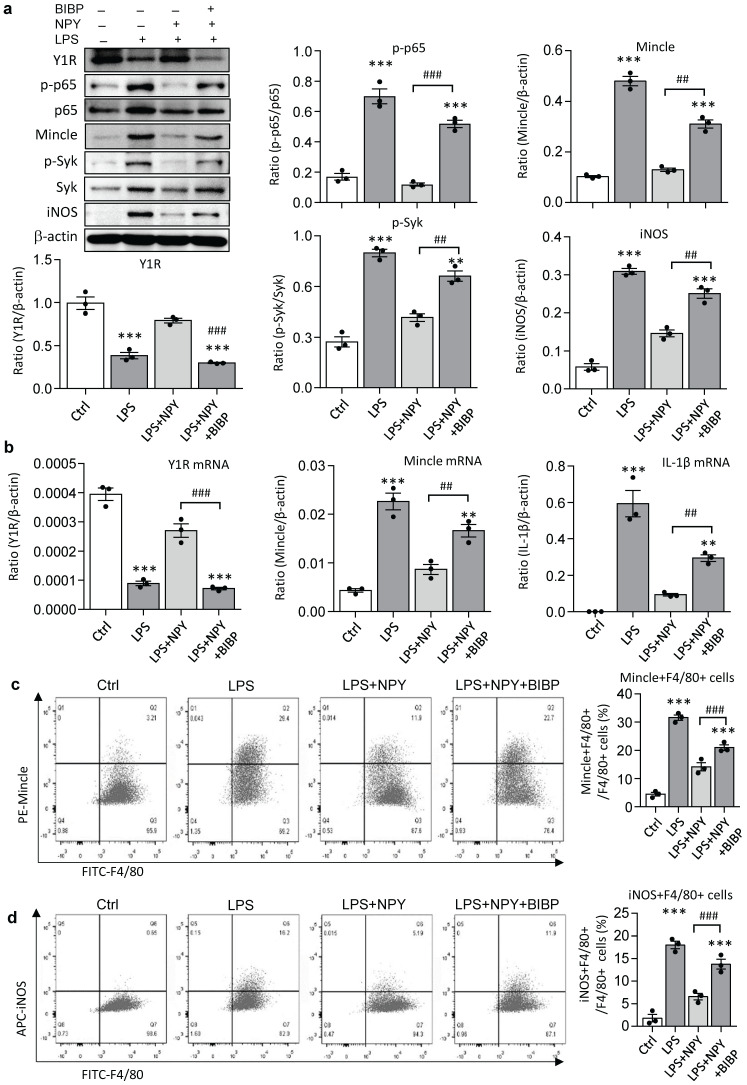
** Blockade of Y1R with BIBP 3226 blunts the inhibitory effect of NPY on LPS-induced NF-κB/p65-Mincle-Syk signaling and M1 macrophage activation by DMBMs *in vitro*. a.** Western blots reveal that addition of BIBP 3226 (300 mg/ml) to BMDMs blocks NPY (200 mg/ml)-induced Y1R but enhances LPS (100 ng/ml)-induced activation of NF-κB/p65-Mincle-Syk signaling and M1 macrophages with high levels of Mincle and iNOS. **b.** Real-time PCR for expression of Y1R, Mincle, and IL-β by BMDMs treated with or without NPY and BIBP 3226. **c, d.** Two-color flow cytometry detects Mincle and iNOS-expressing macrophages (Mincle+iNOS+F4/80+) after treatment with or without NPY and BIBP 3226. Each dot represents one independent experiment, and each bar is the mean ± SEM for 3 independent experiments. BIBP, BIBP3226. ^**^P<0.01, ^***^P<0.001* vs.* Ctrl group;^ #^*P*<0.05, ^##^*P*<0.01, ^###^*P*<0.001 as indicated.

It is reported that NPY signals through the Y1R to inhibit NF-κB signaling and immune response under various disease conditions 35-39. Our recent study also unraveled that NPY acts via Y1R to exert its cardioprotective role in acute myocardial infarction by inhibiting p38/nuclear factor κB (NF-κB)-mediated M1 macrophage activation 35. Thus, we hypothesized that NPY may act via Y1R to exert its renal protective effect on AKI by blocking NF-κB-Mincle-dependent mechanism. This was examined by treating AKI mice with a Y1R antagonist (Y1R) BIBP 3226 (300 μg/kg/day) in the presence of NPY (50 μg/kg/day) i.p. for 3 days. We found that addition of NPY significantly upregulated Y1R while inhibiting cisplatin-induced AKI by blocking NF-κB-Mincle-driven renal inflammation including inactivation of F4/80^+^Mincle^+^iNOS^+^ M1 macrophages (Figures 4 f,g and 5e-h, and Figure 7). In contrast, blockade of Y1R signaling with BIBP 3226 reversed these protective effects of NPY on AKI by largely increasing tubular necrosis and serum levels of creatinine in a dose-dependent manner (Figure 7a, b). Further studies uncovered that treatment with BIBP 3226 blocked NPY-induced expression of Y1R at both mRNA and protein levels, thereby largely enhancing NF-κB-Mincle-Syk signaling and expression of IL-1β, TNFα, and IL-6 in the AKI kidney in a dose-dependent manner (Figure 7 and Supplementary Figure 6). Importantly, flow cytometry revealed that blockade of Y1R with BIBP3226 reversed the inhibitory effect of NPY on activation of Mincle signaling specifically in M1 macrophages in AKI kidneys (Figure 7 c, d). Western blot and real-time PCR analysis further confirmed these notions that treatment with BIBP3226 dose-dependently inhibited NPY-induced Y1R expression, thereby significantly promoting activation of NF-κB-Mincle-Syk signaling and over-expression of proinflammatory cytokines (Figure 7 and Supplementary Figure 6). Similar results were also obtained *in vitro* studies in BMDMs. As shown in Figure 8, western blot, real-time PCR, and flow cytometry analysis detected that addition of BIBP3226 was capable of blocking NPY-Y1R signaling but enhancing LPS-induced NF-κB-Mincle-Syk signaling, therefore resulting in activation of M1 macrophages identified by a marked expression of Mincle, iNOS, IL-1β, TNFα, and IL-6 (Figure 8). Taken together, findings from *in vivo* and *in vitro* studies unraveled that NPY acts via Y1R to inactivate M1 macrophages and protect kidney from AKI by blocking NF-κB-Mincle signaling.

## Discussion

In the present study, we identified that renal resident macrophages as well as peripheral blood and urinary macrophages were a major source of NPY as demonstrated by 70-80% of macrophage population co-expressing NPY markers (F4/80+NPY+). This finding was consistent with the previous notion in various disease conditions 32, 35, 40. However, it was contradictory to the previous report that NPY is expressed by tubular cells 31. Interestingly, we also found that serum levels of NPY were rapidly reduced when AKI developed in both patients and mice. This finding is also discrepancy to the clinical observations in CKD patients in which serum levels of NPY are elevated and correlated with progressive renal injury and the cardiovascular events 24, 26, 27, 29, 30. Although the causes to this discrepancy are largely unknown, it may relate to two possible mechanisms. First, it may be associated with the different kidney environments between AKI and CKD. In CKD patients, the long-term sympathetic hyperactivity as well as hypertension and kidney scarring may stimulate NPY production. In contrast, the AKI patients show necrotic renal inflammation without evidence for NPY production. Second, the source of NPY production may also contribute to this discrepancy. It is well known that the sympathetic nerve hyperactivity is a major source of NPY production in CKD patients 21, 22, 24, 25. In addition, high levels of TGF-b1 from the fibrotic CKD kidney may also stimulate NPY production because TGF-b1 can stimulate NPY production by fibroblasts as we recently reported in a mouse model of cardiac remodeling.^35^ During the high sympathetic-driven conditions in CKD patients 24-30, 41, 42, NPY can be released as a potent vasoconstrictor to cause vascular smooth muscle constriction, resulting in chronic and ischemic renal injury. In contrast, under physiological conditions, macrophages are a rich source of NPY, however, it is lost when macrophages become activated with M1 phenotype during the onset of AKI as observed in the present study. This is also confirmed *in vitro* that NPY was rich in BMDMs but was lost when macrophages became activated with M1 phenotype in response to LPS. It is possible that macrophage-derived NPY may be immunogenic and function as an immunoregulator to inactivate M1 macrophages and inhibit renal necroinflammation during AKI. This is contradictory to the observation in CKD patients in which the long-term sympathetic hyperactivity contributes to an increase in serum NPY levels and progressive renal injury 21, 22, 24, 25, 41, 42. Thus, lowering serum NPY level has been used as a good indicator for the successful treatment with renal denervation in CKD patients with resistant hypertension 43, 44. Taken together, it is likely that sympathetic nerve-derived NPY may be pathogenic in CKD patients by stimulating vasoconstriction, whereas macrophage-derived NPY may be immunogenic and functions as an immunoregulator to protect kidney from AKI by inhibiting M1 macrophages and renal inflammation.

A novel and unexpected finding in the present study was that NPY is renal protective and may have therapeutic potential for AKI. This was supported by the finding that mice null for NPY developed much more severe AKI with worsening tubular necrosis and renal dysfunction. In contrast, consistent with previous studies 33, 34, treatment of AKI mice with exogenous NPY was able to dose-dependently protect kidney from cisplatin-induced AKI, revealing a therapeutic potential of NPY for AKI. These findings were also consistent with our recent report in a mouse model of acute myocardial infarction in which mice lacking NPY develop more severe cardiac injury by increasing the infarcted size with worsening cardiac dysfunction, which is attenuated by treatment with NPY in a dose-dependent manner 35.

It has been well established that NPY acts via Y1R to exert its anti-inflammatory effect by inhibiting NF-κB signaling 35-40. Our recent studies also demonstrated that a transmembrane pattern recognition receptor Mincle (macrophage-inducible C-type lectin, Clec4e) is essential in triggering and maintaining M1 macrophage activation and function, which is tightly regulated by NF-κB signaling 9. Consistent with a known role for NF-κB-Mincle signaling in M1 macrophages-mediated AKI as well as in protumoral activities of tumor-associated macrophages 9-11, 45, the present study unraveled that NPY signals through the Y1R to inactivate M1 macrophages and renal necroinflammation by inhibiting NF-κB-Mincle signaling. We first observed that a loss of NPY in NPY KO mice and BMDMs inhibits Y1R but increase in NF-κB-Mincle signaling, resulting in overactivation of M1 macrophages (F4/80+iNOS+Mincle+ cells) and the development of severe necroinflammation in AKI. In contrast, addition of NPY to the AKI mice and LPS-stimulated BMDMs was capable of dose-dependently upregulating Y1R signaling, thereby blocking NF-κB-Mincle-mediated M1 macrophage activation and proinflammatory cytokines including IL-1β, TNFα, IL-6, and MCP-1, which was reversed by silencing NPY in LPS-stimulated BMDMs. In addition to macrophages, tubular epithelial cells are also highly expressing Y1R 46. As tubular epithelial cells are also a key cell in renal inflammation including AKI 47, it is highly possible that NPY may also bind the Y1R on renal tubular cells to exert its inhibitory effect on NF-kB-driving renal inflammation. This may well explain that loss of NPY in NPY KO mice enhanced while addition of exogenous NPY inhibited tubular necrotic inflammation as seen in this study. Thus, NPY may also bind the Y1R on tubular epithelial cells to suppress NF-kB-mediated renal inflammation, which could be another mechanism through which NPY protects against AKI.

To further validate the regulatory role for NPY-Y1R-NF-κB-Mincle signaling in M1 macrophage activation and AKI, we blocked NPY-Y1R signaling with a Y1R antagonist BIBP3226 in AKI mice and LPS-stimulated BMDMs. As expected, addition of a Y1R antagonist BIBP3226 was able to block NPY-induced upregulation of Y1R signaling, thereby enhancing NF-κB-Mincle-mediated M1 macrophage activation and renal necroinflammation in AKI mice and *in vitro*. The regulatory role for Mincle on M1 macrophage activation was further confirmed by silencing Mincle to inhibit LPS-induced M1 macrophage activation and expression of proinflammatory cytokines.

It should be pointed out that the vasoconstriction caused by NPY may also induce ischemic renal injury, particularly in CKD patients, which challenges the application of NPY in AKI on CKD patients. In addition, although a short-term use of NPY is protective in AKI by inhibiting renal inflammation, the long-term use of NPY could be harmful due to its vasoconstrictive effect on CKD.

## Conclusions

In summary, we identify that NPY is constitutively produced by resident macrophages and functions as a new immunoregulator to inactivate M1 macrophages and inhibit renal necroinflammation under AKI conditions. Importantly, NPY is renoprotective and has therapeutic effect on AKI. Mechanistically, NPY signals through the Y1R to exert its inhibitory effect on M1 macrophage-mediated AKI by blocking NF-κB-Mincle signaling.

## Methods

### Blood samples from AKI patients

Total of 15 AKI patients and 6 health individuals from the Department of Nephrology, Affiliated Traditional Chinese Medicine Hospital of Southwest Medical University were enrolled into this study. The blood samples were collected from AKI patients at days 3-5 after diagnosis and serum was separated and stored at -80C for measuring NPY by an ELISA kit (R&D, Minneapolis, MN) The clinical characteristics of AKI patients were shown in the Supplementary Table 1. Of them, 5 AKI patients were sepsis-related; 8 cases were associated with ischemia; and 2 cases were related to drugs toxicity. In addition, blood mononuclear cells from groups of 6 AKI patients and health controls were obtained by using Ficoll-Pague density gradient centrifugation and urinary macrophages were collected from the urine sediments. A written informed consent was given to all patients and the studies were carried out according to the guidelines approved by the Ethics Committee of Affiliated Traditional Chinese Medicine Hospital of Southwest Medical University (approval number: KY2019057).

### NPY KO mice and animal model of AKI

The 129S-Npy^tm1Rpa^/J NPY WT/KO mice (stock number 004545) were purchased from the Jackson Laboratory (Bar Habor, ME). Mice deficient for NPY show normal phenotype with normal food intake and body weight although occasionally mild seizures occur in NPY-deficient mice 48. Genotypes of NPY WT or KO mice were determined in tail DNA samples using primers as previously described.^35^ The AKI mouse model was induced in groups of 8-10 NPY KO or WT mice by a single dose of cisplatin at 20mg/kg body via intraperitoneal injection at day 0 and sacrificed on days 1, 2, and 3 as previously described 8-11. To explore the role and therapeutic effect of NPY on cisplatin-induced AKI, immediately after administration of cisplatin, groups of 6-8 NPY WT mice were given with exogenous NPY (Tocris Bioscience, Bristol, UK) at dosages of 0, 25, 50, and 100 μg/kg body weight or saline control via daily i.p. till being sacrificed on day 3. To investigate the signaling mechanism of NPY via the Y1R, 30 min after receiving NPY treatment, groups of 6-8 AKI NPY WT mice were treated with a Y1R antagonist BIBP 3226 (Santa Cruz Biotechnology, Dallas, Texas) at dosages of 0, 100, and 300 μg/kg via daily i.p. till being sacrificed on day 3. Control AKI mice had the same treatment protocol but received saline instead of BIBP 3226. All experimental protocols were approved by the Animal Experimentation Ethics Committee, The Chinese University of Hong Kong.

### Renal Function and Histology

Serum levels of creatinine were determined by using the quantitative enzymatic method of the Creatinine LiquiColor Test kit (Stanbio Laboratory, Texas) according to the manufacturer's protocol, while blood urea nitrogen (BUN) was measured by using BUN Assay kit (Jiancheng bio, Nanjing, China). Kidney tissues were fixed with Histochoice Tissue Fixation MB (AMRESCO, VWR Life Science, PA) and tubular necrosis was examined in paraffin sections (3 µm) by hematoxylin and Eosin (HE) or Periodic Acid-Schiff (PAS) staining (Sigma, St. Louis, MO). A total of 500 cortical tubules were examined and the numbers of tubules with necrosis was counted and expressed as percentage as previously described 8-11.

### Immunohistochemistry

Immunohistochemistry was performed in paraffin sections with antibodies against NPY (Cell Signaling Technology, MA) or macrophages (CD68 or F4/80, Bio-Rad, Hercules, CA) by using a microwave-based antigen-retrieval method as previously described 8-11. Two-color immunofluorescence was performed in snap-frozen tissue sections or cell-spots to detect NPY-producing macrophages with PE anti-mouse F4/80 antibody (BioLegend, San Diego, CA) and Alexa 488-conjugated NPY (Santa Cruz). After being stained, sections were counterstained with hematoxylin or DAPI and examined under a fluorescent microscope (Leica, Wetzlar, Germany). Positive cells were counted under 10 high-power fields (HF, ×40) per section and expressed to cells/HF.

### Genetically modified BMDMs *in vitro*

To silence NPY or Mincle, bone marrow-derived macrophages (BMDMs) were incubated with Lipofectamine™ RNAiMAX and siRNA-NPY or siRNA-Mincle respectively for 24 hours, followed by stimulation with LPS (100ng/ml) for 24 hours for examining the diverse effect of silencing NPY or Mincle on activation of M1 macrophages. The sequence of siRNA-NPY is sense: CCAAGCGAAUCAACAUCAU, antisense: AUGAUGUUGAUUCGCUUGG; while sequence of siRNA-Mincle is sense: CCUUUGAACUGGAAACAUUTT, antisense: AAUGUUUCCAGUUCAAAGGTT.

### Cell culture

BMDMs were isolated from NPY WT or KO mice by flushing the femur and tibia with DMEM and cultured for 7 days with DMEM containing 10% fetal bovine serum (FBS; ExCell Bio) and 50 ng/ml macrophage colony stimulating factor (M-CSF) to obtain more than 95% of F4/80+ macrophages. The role and signaling mechanisms of NPY in inactivating M1 macrophages via the Y1R-NF-κB-Mincle pathway were studied in BMDMs with or without NPY KO, siNPY or siMincle. Cells were cultured for 0, 1, 3, 6, 12, and 24 hours in the presence or absence of LPS (100ng/ml, Sigma-Aldrich, St. Louis, MO) and exogenous NPY (0, 100, 200 mg/ml) with or without Y1R antagonist BIBP (0,100, 300 mg/ml). The phosphorylation of NF-κB/p65 and expression of Y1R, Mincle, iNOS, IL-6, IL-1β, and TNFα were examined by western blot and real-time PCR.

### Western blotting

Total protein from cells and kidney were isolated by RIPA buffer for western blot analysis as previously described 8-11, 35. Primary antibodies against NPY, Y1R, phospho (p)-NF-κB/p-p65, NF-κB/p65, p-Syk (all from Cell Signaling), iNOS (Abcam, Cambridge, UK), Mincle (Santa Cruz or MBL International, Woburn, MA), and β-actin (Santa Cruz) were used. After being stained and washed, the membranes were incubated with LI-COR IRDye 800 conjugated secondary antibodies (Rockland Immunochemicals, Gilbertsville, PA) in the dark for 1 hour. Signals were then scanned and visualized by the Odyssey Infrared Imaging System (Li-COR Biosciences, Lincoln, NE). The ratio of the targeted protein was subjected to β-actin and quantified with ImageJ software (NIH, Bethesda, MD).

### Real-time PCR

Total RNA was isolated from kidney and cells by using TRIzol Reagent (TianGen, China) and reversely transcribed to cDNA with a Reverse Transcription Kit (Promega, China). The mRNA expression of NPY, Y1R, Mincle, IL-1β, IL-6, TNF-α and iNOS were quantified by using Bio-Rad iQ SYBR Green supermix with the Opticon2 (Bio-Rad). The primers used in this study have described previously 8-11, 35.

### Flow cytometry

Mouse kidney was digested into a single cell suspension with Blenzyme 4 (Roche, Indianapolis, IN, USA) at 37 °C for 30 minutes followed by sieving. Then cells from the mouse kidney or peripheral blood of health or AKI patients were fixed for 30 min in IC Fixation Buffer (eBioscience), permeabilized in permeabilization buffer (eBioscience) for 15 min at room temperature, and then labeled with the combinations of the following antibodies including APC-conjugated iNOS (eBioscience), FITC-F4/80 (BioLegend) or ERM1 (recognized human macrophage F4/80 antigen, Santa Cru), APC-Mincle (Santa Crus), PE-NPY (Santa Cruz) for overnight at 4 °C. After being washed, cells were gated and analyzed by the FACSCalibur flow cytometer (BD Biosciences, San Jose, CA, USA).

### ELISA

The concentration of NPY in patient and mouse serum was measured by Enzyme‐linked immunosorbent assay using the Quantikine ELISA Kits (R&D, Minneapolis, MN) according to the product protocols.

### Statistical Analysis

All the data of this study were shown as mean ± SD. Statistical analyses were performed using one-way ANOVA, followed by Newman-Keuls posttest using a SPSS 21.0 Software (SPSS, Inc., USA). *P<*0.05 was means to be a statistical difference.

## Supplementary Material

Supplementary figures and tables.

## Abbreviations

AKI: acute kidney injury
NPY: neuropeptide Y
Y1R: NPY receptor 1
Mincle: macrophage-inducible c-type lectin
NF-κB: nuclear factor kappa-B
CKD: chronic kidney disease
PYY: peptide YY
PP: pancreatic polypeptide
KO: knock-out
WT: wild-type
ELISA: enzyme linked immunosorbent assay
IL-1β: interleukin 1β
IL-6: interleukin 6
TNFα: tumor necrosis factor α
MCP-1(Ccl2): chemokine (C-C motif) ligand 2
BUN: blood urea nitrogen
PCR: polymerase chain reaction
BMDMs: bone marrow-derived macrophages
LPS: Lipopolysaccharide
SYK: spleen tyrosine kinase
CIS: cisplatin
IRI: ischemia-reperfusion injury
HE: hematoxylin and eosin
PAS: periodic acid-schiff
