# Supplementary material for: Neuropeptide Y protects kidney from acute kidney injury by inactivating M1 macrophages via the Y1R-NF-κB-Mincle-dependent mechanism
Source: Int J Biol Sci. 2023 Jan 1;19(2):521–36. doi: 10.7150/ijbs.80200 (PMC9830509; doi:10.7150/ijbs.80200)
Supplement: Supplementary file 1 — Supplementary figures and tables. [file ijbsv19p0521s1.pdf]

Supplementary Table 1. The clinical characteristics of AKI patients and health individuals

AKI patients					
**No**	Gender	Age	Creatinine (μmol/L)	GFR (ml/min)	Disease
**1**	Female	17	211	28.9	Ischemic AKI
**2**	Female	28	188.3	30.7	Ischemic AKI
**3**	Male	52	345.7	16.9	Septic AKI
**4**	Male	69	175.7	33.9	Drug toxicity AKI
**5**	Male	51	720	5.2	Drug toxicity AKI
**6**	Male	21	138.5	44.3	Septic AKI
**7**	Female	19	194	31.5	Ischemic AKI
**8**	male	62	218.8	27.3	Septic AKI
**9**	Female	21	158	39.7	Ischemic AKI
**10**	Male	63	253	22.7	Ischemic AKI
**11**	Female	55	234	20	Septic AKI
**12**	Male	50	397.5	16.5	Septic AKI
**13**	Female	40	197.2	26.7	Ischemic AKI
**14**	Male	29	645	9.3	Ischemic AKI
**15**	Female	66	165.7	36.4	Ischemic AKI
	Mean±SEM	44.6±4.46	284.27±43.91	26±2.74	
**Health people**					
**No**	Gender	Age	Creatinine (μmol/L)	GFR (ml/min)	
**1**	Male	54	89.4	85.3	
**2**	Female	51	50.4	108.1	
**3**	Male	22	52.2	130.1	
**4**	Male	26	47.9	130.1	
**5**	Female	23	59.7	123.6	
**6**	Female	22	59.6	138.5	
	Mean±SEM	33±6.21	59.87±6.23	119.28±7.96	

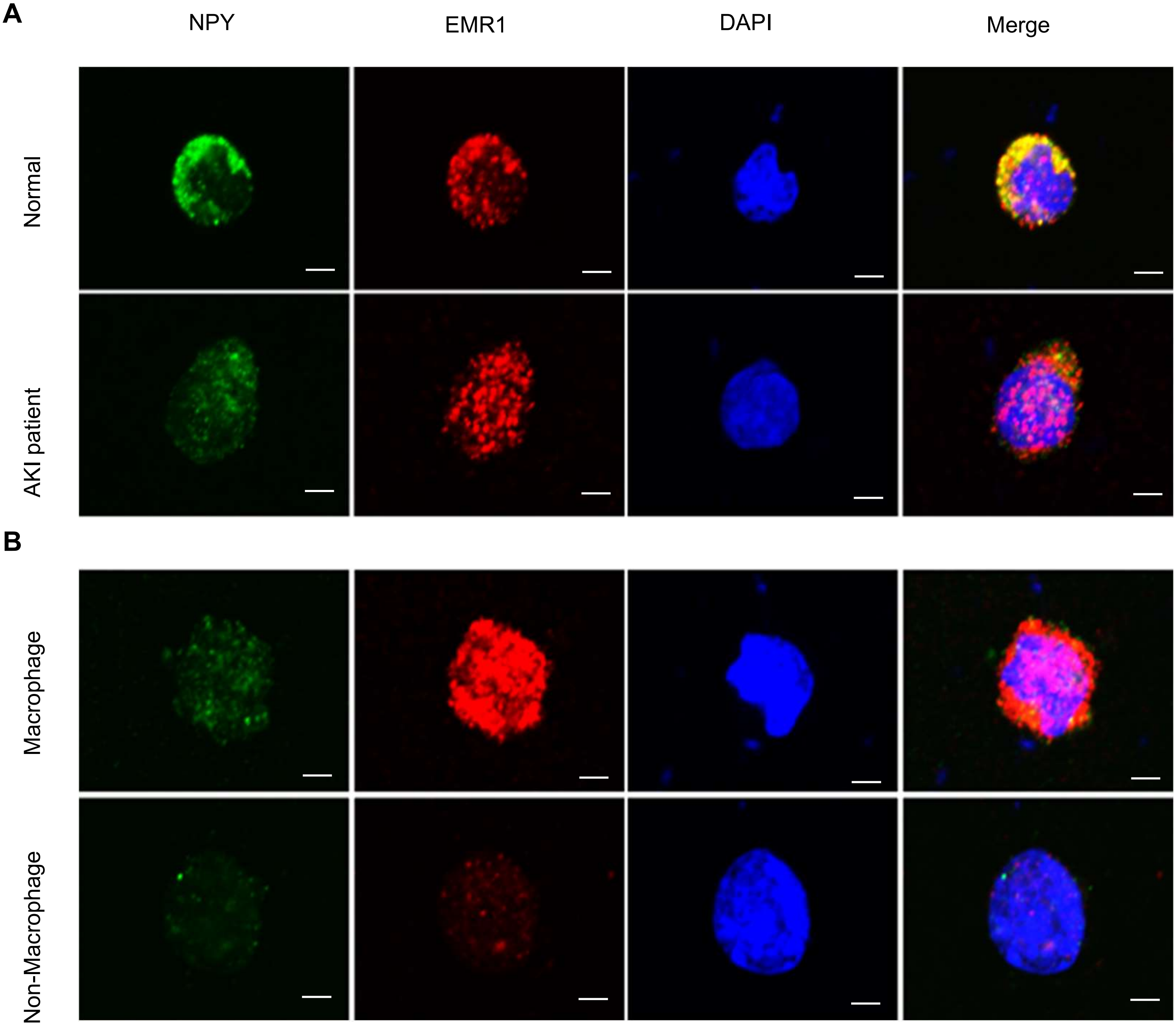

Supplementary Figure 1. Tow-color immunofluorescence shows that NPY is localized within cytoplasm of peripheral blood monocytes (A) and urinary macrophages (B) and is abundant in normal population but lost in patients with AKI. Note that NPY is not detectable in other cell population (B). Data represent groups of 6 health or AKI patients. Scale bar, 100 μl.

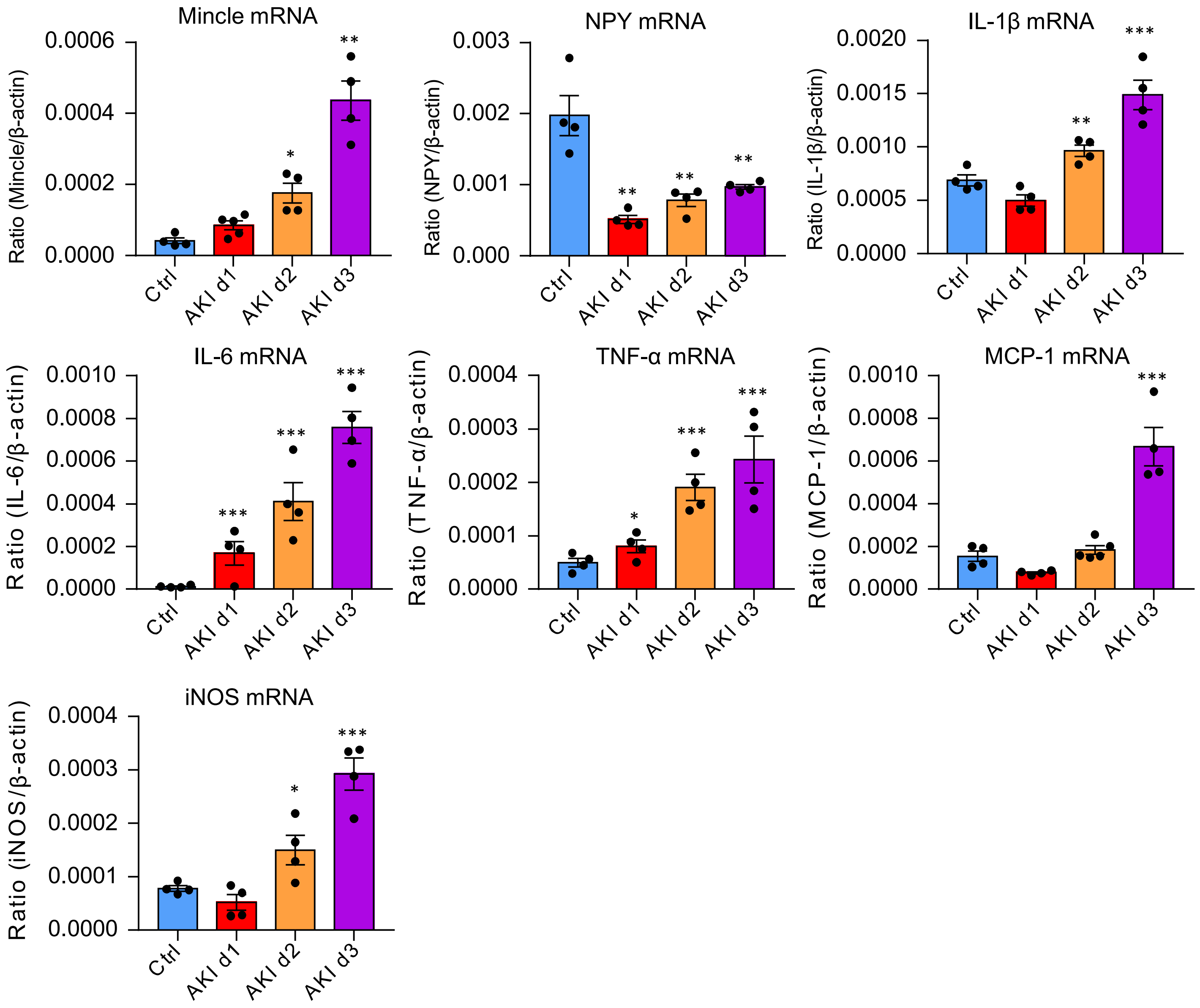

Supplementary Figure 2. Real-time PCR shows that loss of renal NPY in cisplatin-induced AKI kidneys is associated with a marked increase in expression of Mincle, IL-1β, TNFα, IL-6, MCP-1, and iNOS in a time-dependent manner. Each bar represents mean±SEM for groups of 6 mice. *P<0.05, **P<0.01, P<0.001 compared to normal control mice.

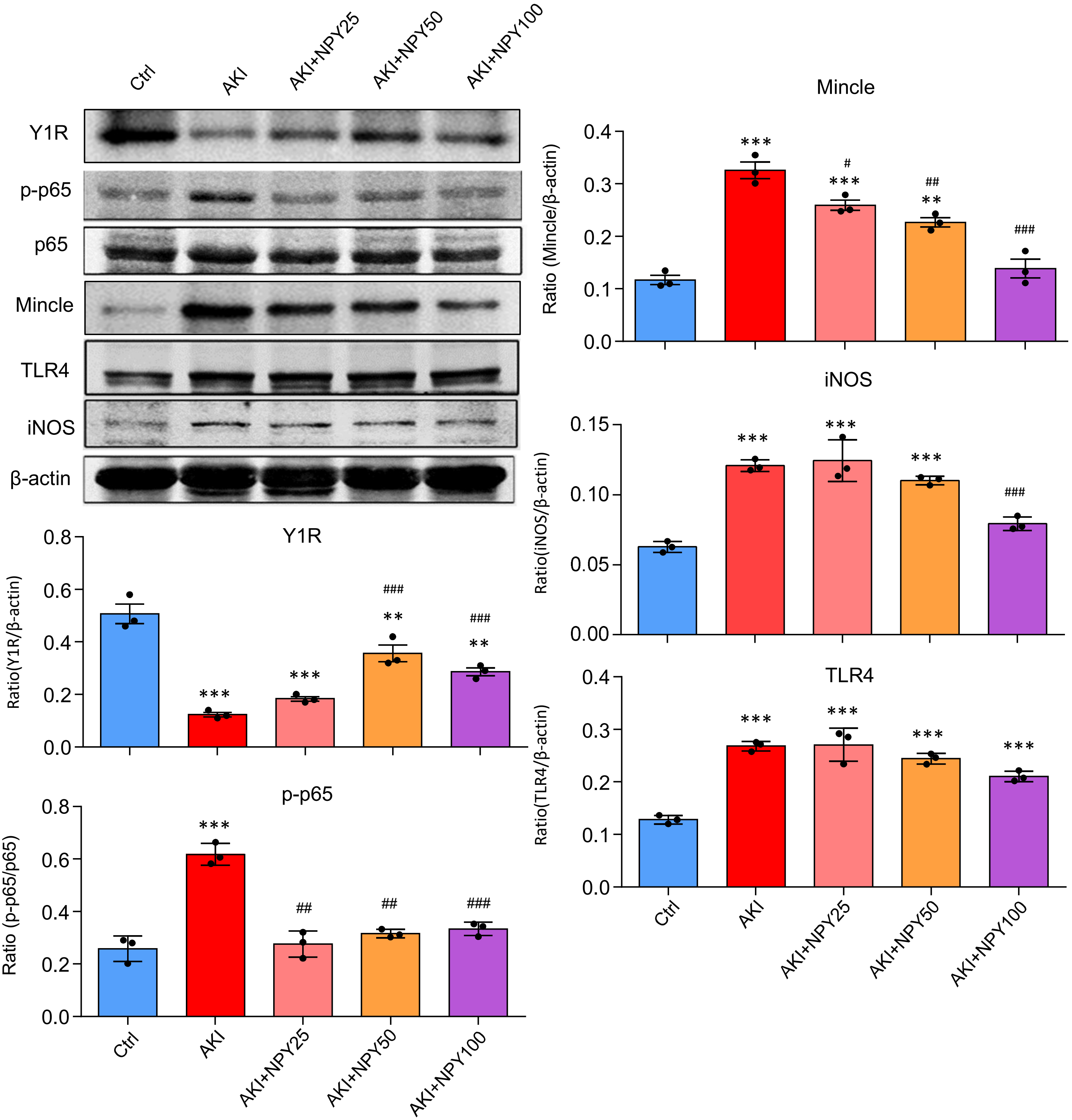

Supplementary Figure 3. Western blot analysis shows that treatment of cisplatin-induced AKI mice with exogenous NPY dose-dependently upregulates Y1R and inhibits NF-κB signaling, thereby blocking expression of Mincle and iNOS without alteration of TLR4 in the AKI kidney. Each bar represents mean ± SEM for groups of 6 mice. *P<0.05, **P<0.01, P<0.001 compared to normal control mice; #P<0.05, ##P<0.01, ###P<0.001 compared with untreated AKI.

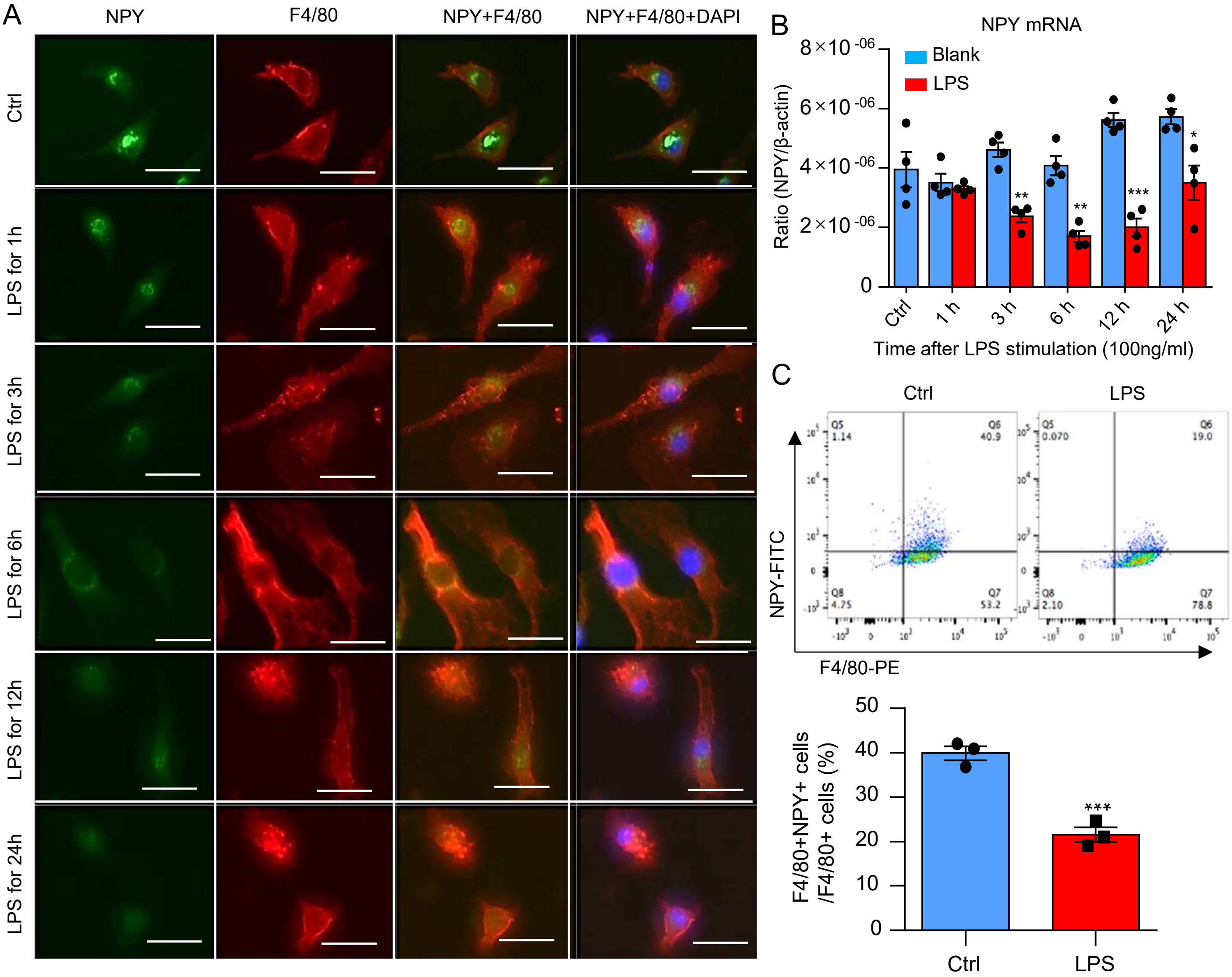

Supplementary Figure 4. NPY is located in cytoplasm of BMDM and is reduced by LPS in time-dependent pattern. BMDMs were cultured with LPS (300μg/ml) and NPY expression by macrophages was examined by two-color immunofluorescence, real-time PCR, and flow cytometry. Data represent mean±SEM for three independent experiments. *, p<0.05, **p<0.01, ***p<0.001 compared to the control (Ctrl). Sale bar, 100 μl.

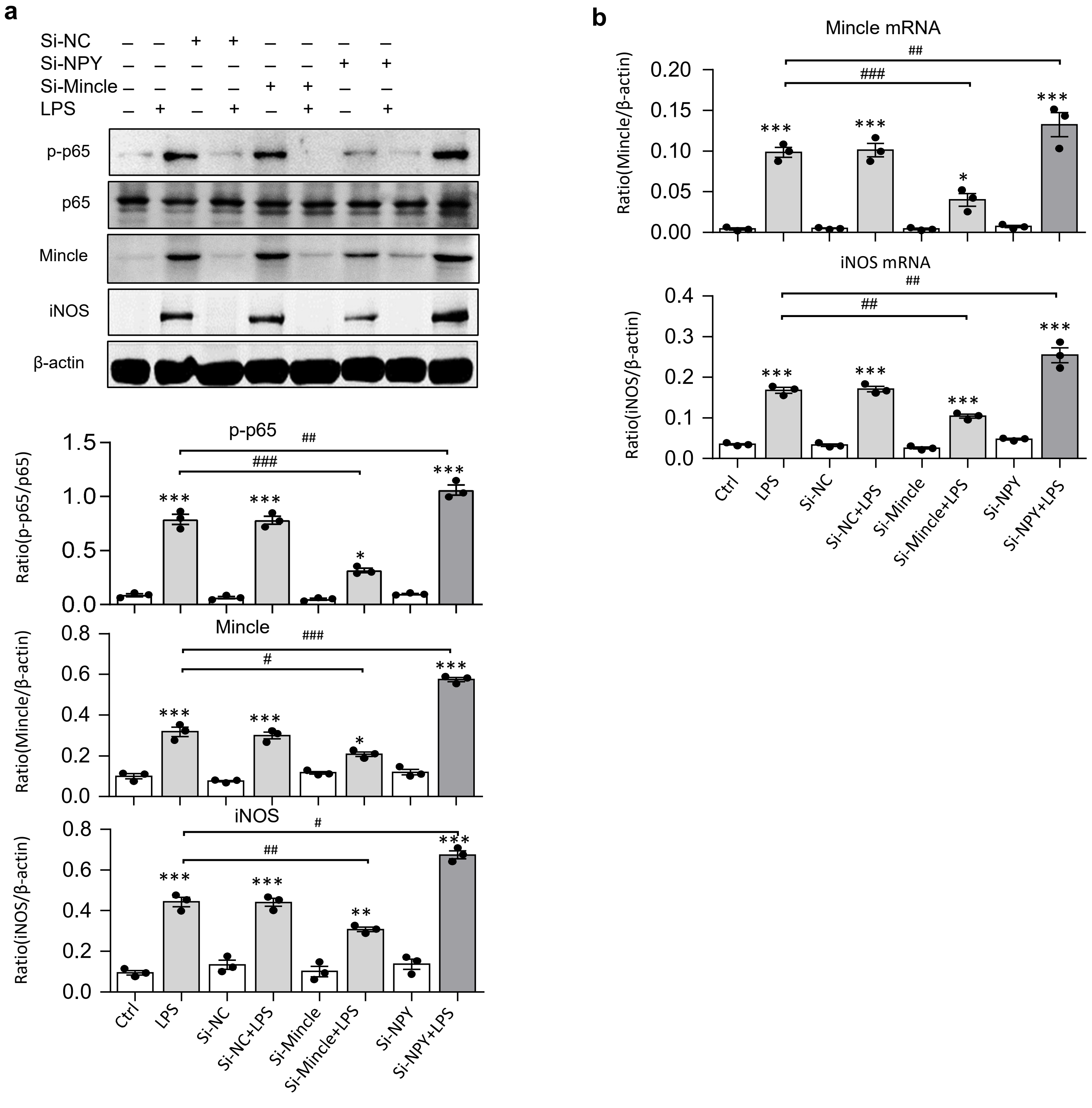

Supplementary Figure 5. Silencing NPY in BMDMs largely enhances LPS-induced activation of NF-κB/p65-Mincle signaling, which is reversed by silencing Mincle in vitro.*, p<0.05, **p<0.01, ***p<0.001 compared to the control (Ctrl). $^{\#}$P<0.05, $^{\#\#}$P<0.01, $^{\#\#\#}$P<0.001 compared with LPS.

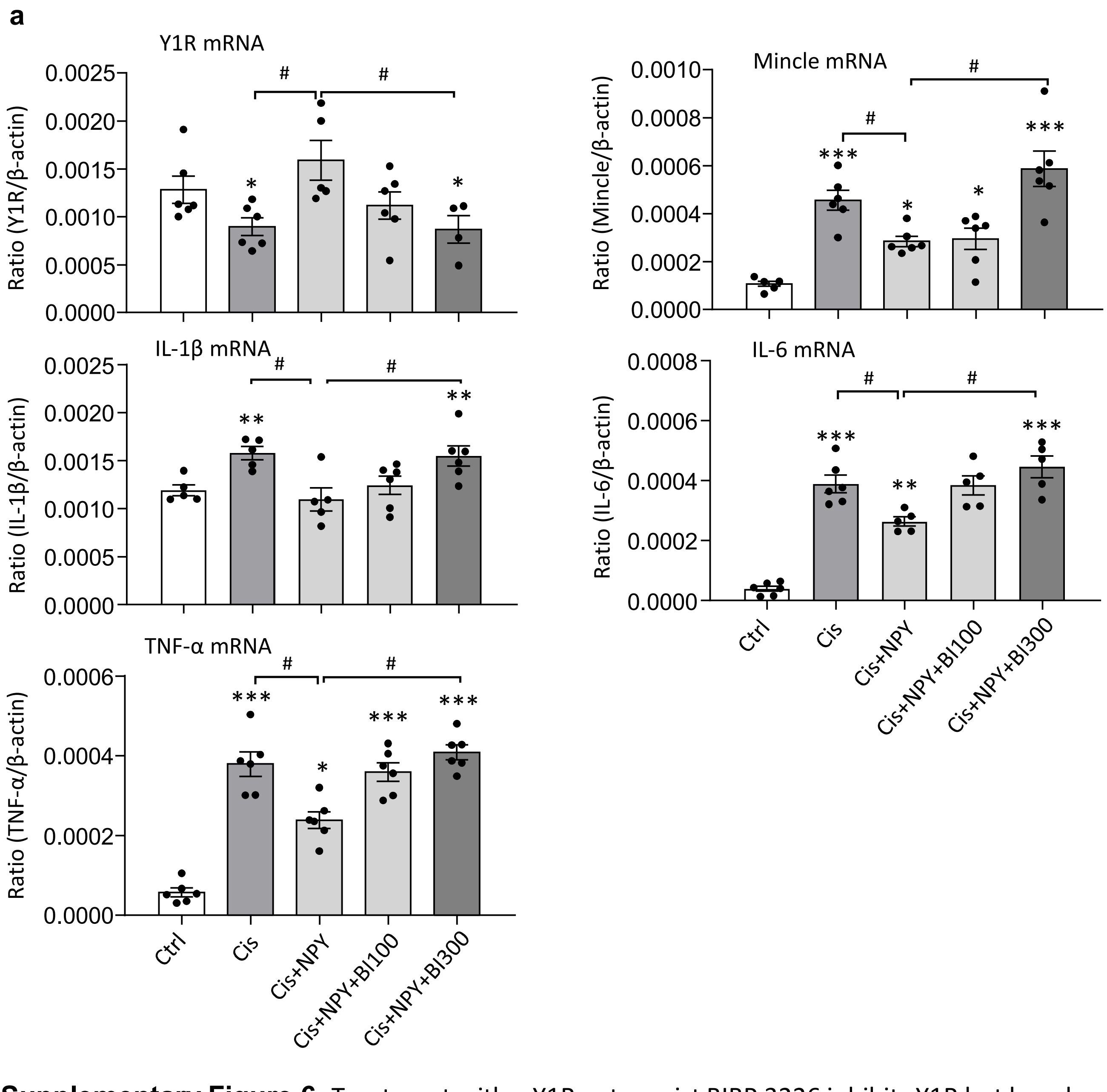

Supplementary Figure 6. Treatment with a Y1R antagonist BIBP 3226 inhibits Y1R but largely enhances Mincle, IL-6, IL-1β, and TNF-α mRNA expression in cisplatin-induced AKI in mice.*, p<0.05, **p<0.01, ***p<0.001 compared to the control (Ctrl). #P<0.05 as indicated.
